# Brain Connectivity Studies in Schizophrenia: Unravelling the Effects of Antipsychotics

**DOI:** 10.2174/157015912803217305

**Published:** 2012-09

**Authors:** Ayna B Nejad, Bjørn H Ebdrup, Birte Y Glenthøj, Hartwig R Siebner

**Affiliations:** 1 Danish Research Centre for Magnetic Resonance, Copenhagen University Hospital, Hvidovre, Denmark; 2 Center for Neuropsychiatric Schizophrenia Research & Center for Clinical Intervention and Neuropsychiatric Schizophrenia Research, Copenhagen University Hospital, Psychiatric Center Glostrup, Denmark; 3 Department of Neurology, Psychiatry and Sensory Sciences, Faculty of Health Sciences, University of Copenhagen, Denmark

**Keywords:** Antipsychotic drug treatment, brain connectivity, fMRI, schizophrenia.

## Abstract

Impaired brain connectivity is a hallmark of schizophrenia brain dysfunction. However, the effect of drug treatment and challenges on the dysconnectivity of functional networks in schizophrenia is an understudied area. In this review, we provide an overview of functional magnetic resonance imaging studies examining dysconnectivity in schizophrenia and discuss the few studies which have also attempted to probe connectivity changes with antipsychotic drug treatment. We conclude with a discussion of possible avenues for further investigation.

## INTRODUCTION

Schizophrenia is a severe and heterogeneous brain disease with onset usually in late adolescence or early adulthood. Symptoms are typically classified as positive (e.g. hallucinations, delusions and thought disorder) or negative (e.g. affective flattening, poverty of speech and anhedonia) [1]. Also, cognitive deficits are widespread and become evident even in prodromal phases of psychosis [2]. Relatively little is known about the aetiology of schizophrenia but it is believed to be associated with aberrant neurodevelopment that is influenced by genetic liability and environmental factors [3]. 

Functional neuroimaging has been widely used to pinpoint the neural mechanisms underlying the core symptoms of schizophrenia. Early neuroimaging studies have tried to identify abnormal task-related activation patterns in localised brain areas that may underlie the symptoms of schizophrenia [4]. A particular focus has been placed upon the prefrontal cortex which is implicated in many aspects of executive function. While early studies found prefrontal areas to be hypoactive in patients with schizophrenia [5-7], prefrontal activity changes have been variable across subsequent studies with some reporting prefrontal hyperactivation compared to healthy control subjects [8]. These prefrontal activity changes have been difficult to interpret. Prefrontal hyperactivity might indicate inefficient neural processing, or compensatory activity for a dysfunction elsewhere in the brain. Likewise, hypofrontality might indicate the inability to sufficiently activate a prefrontal region during an experimental task, or might be attributed to a reactive engagement of alternative neural strategies, which do not involve the prefrontal cortex [9]. Moreover, there is no consistent evidence indicating clear hypo- or hyperactivations in schizophrenia. Heterogeneity across individual studies can originate from different sources for example performance differences but also illness chronicity and exposure to antipsychotic medication. 

**Fig. (1):**
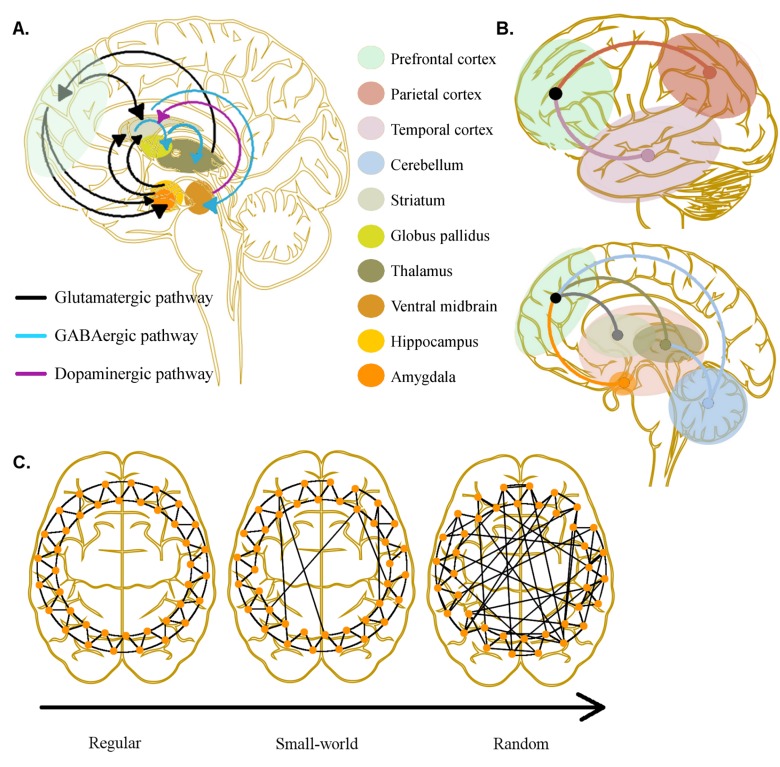
Dysconnectivity in schizophrenia. **A**) Pathways between subcortical and prefrontal cortical regions: A specific pathogenetic pathway has yet to be identified in schizophrenia but disruption in one may have implications to widespread functioning due to feedback loops. Generally, the purported increased dopaminergic activity in striatum could underlie positive symptoms and has implications for prefrontal cortical function leading to cognitive deficits. **B**) Dysconnectivity in schizophrenia studied with fMRI: Dysfunctional interactions among prefrontal, temporal and parietal cortices have been found as well as between prefrontal cortex, striatum, thalamus, amygdala and cerebellum. These have often been studied with functional connectivity but effective connectivity has also been applied modelling the directionality of connections between regions. **C**) Dysconnectivity from a global perspective: Topology of brain connectivity can be studied with graph theory where a disruption of ’small-world’ properties has been detected in schizophrenia. A highly regular network topology has short path lengths and high degree of connectivity with nearest neighbours whereas random graphs have long path lengths and low clustering. A small-world network lies in between these where high clustering and a few long-range connections increases cost-effectiveness of communication within whole-brain networks (see text for references).

The circuits thought to be involved in schizophrenia are complex, involving various neurotransmitters and receptors at the cortical and subcortical levels (Fig. **1a**). However, antipsychotic medications, most with complex receptor binding profiles, all share an affinity for the D_2_ receptor. There is recent meta-analytical evidence indicating a 14% increase of striatal presynaptic dopaminergic neurotransmission in schizophrenia patients as compared to controls [13, 14]. Increased dopaminergic activity in the striatum [15] as well as frontal D_2_ receptor binding potentials [16] have been linked to the manifestation of positive symptoms in schizophrenia [17], whereas an imbalance of prefrontal dopamine D_1_ to D_2_ receptor signalling has been implicated in the manifestation of positive as well as negative and cognitive symptoms in schizophrenia [18, 19]. Because of their strong striatal D_2_ antagonism, the first generation of antipsychotics (FGAs) produced extrapyramidal side effects. However, second generation antipsychotics (SGAs) generally have a therapeutic effect at doses with lower striatal D_2_ receptor blockade and therefore produce less extrapyramidal side effects. It was initially proposed that SGAs would be superior in targeting negative symptoms and cognitive deficits. The beneficial effect on negative and cognitive symptoms was thought to be mediated by an antagonistic effect of some SGAs on serotoninergic neurotransmission [20] which enhanced the dopamine efflux in the prefrontal cortex in animal studies [21, 22]. This prefrontal dopamine efflux together with a weak D_2_ antagonism might thus improve cognitive deficits by restoring the D_2_:D_1_ receptor signalling ratio which has been proposed to be integral for efficient cognitive processing of external stimuli [23, 24]. Yet, large well-designed studies comparing FGA and SGA treatments have not found better clinical efficacy of SGAs in targeting negative symptoms and cognitive deficits [25]. 

Neuroimaging studies in schizophrenia have also focused on localised brain activity in asking whether antipsychotic medication is able to normalise aberrant brain activity [10-12]. Many studies have shown that antipsychotic treatment normalises hypoactivation of lateral prefrontal regions to the activity level of healthy control subjects [26-31]. Again, the findings have not always been consistent. In one study, medication-naïve patients were scanned before and after ten weeks of SGA treatment which resulted in no change in brain activity [32]. A cross-sectional study found widespread brain activity to be increased in an antipsychotic-naïve group of schizophrenia patients compared to control subjects but no differences were found between SGA-treated patients and control subjects [33]. One study did not find modulation of the lateral prefrontal cortex with antipsychotic treatment but rather of the anterior cingulate cortex when subjects performed a motor response task [34]. Another question which has attracted considerable attention is whether SGAs, are superior in improving neural efficiency compared to FGAs. There have been studies purporting a greater increase in lateral prefrontal and anterior cingulate cortical activity in patients treated with SGAs compared to those treated with FGAs [35-38], yet again this has not always been found [39]. 

A focus on ‘localisation of dysfunction’ has been replaced in recent years by an ‘impaired connectivity’ perspective which postulates that impaired functional interactions between brain regions, rather than changes in regional neuronal activity of specific cortical areas, underlie the dysfunction associated with schizophrenia [40, 41]. Dysfunctional connectivity of brain signalling pathways is thought to emerge during neurodevelopment [42, 43]. In accordance with this notion, many of the genetic polymorphisms related to risk for schizophrenia are associated with axonal guidance, synaptic architecture and plasticity [44]. Histological findings have suggested that schizophrenic brains possess necrotic oligodendrocytes and less dendritic arborisation [45, 46] implying impaired anatomical connectivity. With respects to functional impairment, dopamine dysregulation has been suggested to be secondary to N-methyl-D-aspartate (NMDA) receptor hypofunction in schizophrenia [47, 48]. Prefrontal NMDA hypofunction has been found to hyperstimulate D_2_ receptors in the striatum which in turn, *via *striatal gamma-aminobutyric acid (GABA) interneurons, can further weaken cortical glutamatergic transmission [42, 47]. Such dysfunctions at the synaptic level are thought to impair the communication between neurons which form functional circuits resulting in dysconnectivity at the systems level. An antagonistic effect on D_2_ receptors might therefore promote more efficient connectivity between subcortical and cortical regions. Indeed, animal models of schizophrenia have revealed that pharmacologically-induced cortical desynchronisation can be reversed by FGAs and SGAs [49-51]. 

Numerous neuroimaging studies in humans have provided convergent evidence that in schizophrenia, functional brain networks express abnormal connectivity patterns both at rest and during experimental tasks [40, 52-56]. Functional connectivity refers to the temporal coupling of regional neural activity among brain areas. It reflects the amount of shared changes in activity among brain regions without making assumptions about the underlying pathways, structure or causality. Effective connectivity describes neural connectivity in brain networks in terms of directional and context-dependent effects of one brain region on another. Hence, effective connectivity analyses make inferences regarding the information flow between brain sites [57]. Effective connectivity is being increasingly assessed through statistical methods such as psychophysiological interactions, structural equation modelling, dynamic causal modelling and Granger causality.

Functional neuroimaging with positron emission tomography (PET) and functional magnetic resonance imaging (fMRI) techniques can be used to study whole-brain functional and effective connectivity in schizophrenia. In recent years, fMRI has gained popularity over PET imaging as it is the less expensive and invasive choice of the two. Most fMRI connectivity studies use the blood oxygen level dependent (BOLD) signal as a neurovascular index of regional synaptic activity [58]. BOLD fMRI offers good spatial resolution and whole-brain coverage, but has limited temporal resolution. In contrast, electroencephalography (EEG), magnetoencephalography (MEG) and transcranial magnetic stimulation (TMS) are either measuring (EEG, MEG) or interfering (TMS) with regional neural activity offering excellent temporal resolution in the millisecond range. The drawback of these methods is that they mainly capture cortical activity and spatial resolution is limited [59]. Therefore, fMRI and EEG/MEG/TMS nicely complement each other in terms of temporal and spatial assessment of functional brain connectivity. Although all these method can be used to assess functional and effective connectivity within the brain, studies observing abnormal cortico-subcortical connectivity have mainly used fMRI since regional activity in deep grey matter nuclei cannot be assessed with EEG, MEG or TMS. In this review the focus will be made on fMRI studies although there have been a few notable EEG studies observing widespread cortical connectivity changes with antipsychotic treatment in schizophrenia [60-62]. 

Brain imaging studies have also employed methods which capture the distributed patterns of neural activity in brain networks rather than the coupling of activity changes in focal regions. Much of this work, but not all, is based on data collected while participants are at rest. Resting state networks have been found replicable across subjects and over time, and spatially overlap networks which are found in activation studies so therefore seem functionally meaningful [63]. Multivariate methods such as independent component analysis, partial least squares analysis and graph theoretical methods have been used to assess changes in functional networks of schizophrenia patients, and point to a distributed inefficiency of large-scale cortical communication in patients.

Although abnormal brain connectivity has been emphasised as a critical aspect of the pathophysiology of schizophrenia, only a few pharmacological neuroimaging studies have concentrated on the functional impact of psychopharmacological treatment on brain connectivity in patients with schizophrenia. This is surprising as pharmacologically-induced increases or decreases in regional neural activity do not necessarily imply that dysconnectivity has also been ameliorated. Here we will review the potential effect of antipsychotic medications on the brain connectivity of patients affected with psychosis. In order to review the literature, we will summarise abnormal connectivity patterns which, where available, have been identified in samples minimally affected by medication (drug-naïve or first-episode psychosis patients, prodromal individuals, and healthy siblings of schizophrenia patients who are at high genetic risk for the disorder). We will summarise dysconnectivity of local circuits as well as distributed or topological differences in schizophrenia patients and relate these findings to reports of changes in connectivity induced by pharmacological challenges or interventions (Fig. **1b**). Based on these considerations, we comment on future perspectives for neuroimaging research to unravel the impact of pharmacological interventions on impaired connectivity in schizophrenia. 

## DYSCONNECTIVITY IN THE CORTICO-SUBCORTICAL CIRCUIT

Dopaminergic hyperactivity within the striatum has often been regarded as a core feature of schizophrenia and seems to predate the onset of illness. One way to test this is to study subjects at ultra high clinical risk of psychosis. Compared to the general population with a lifetime risk of 1%, these individuals have a much higher risk of developing a schizophrenia spectrum disorder [64], ranging from 18% after six months up to 36% after three years [65]. The clinical high risk state for psychosis is also characterized by significant cognitive impairments [66] and underlying neurobiological abnormalities in the structure [67-69], function [70-72], connectivity [73], and neurochemistry [64, 74, 75] of the brain. There is particular evidence that increased synthesis capacity of dopamine in the striatum, as assessed by PET fluorodopa binding, is associated with hypofrontality during working memory and verbal fluency tasks, assessed by fMRI [70, 71]. Cognitive deficits, such as working memory deficits, in patients with schizophrenia have been proposed to result from an unstable prefrontal cortical state susceptible to irrelevant distractors and linked to activity in the striatum [76]. Tan *et al.* [77] found a reduced frontostriatal inhibitory interaction in carriers of risk-for-schizophrenia alleles of the RAC-alpha serine/threonine-protein kinase (Akt1) gene and the dopamine receptor D2 (DRD2) gene during manipulation processes of working memory. The study further found that schizophrenia patients carrying risk alleles of Akt1 and DRD2 genes which influence D_2_ receptor function had less illness-related reductions in IQ levels with higher doses of antipsychotics than non-risk allele carriers. The findings underline the importance of frontostriatal interactions in general cognitive abilities, and the impact gene-drug interactions might have on connectivity and behavioural response to treatment.

Aberrant reward processing and striatal hyperdopaminergic activity has been theorized to be linked to the development of positive symptoms [17] in that many symptoms seem to be linked to misplaced salience on irrelevant stimuli leading to delusional thought and hallucinations. Striatal BOLD activity during reward processing has been recently linked to positive symptoms in an antipsychotic-naïve patient sample [79]. Drug-naive (n=8) and drug-free (n=7) patients with schizophrenia showed reduced functional connectivity between the medial prefrontal cortex and ventral striatum during processing of reward and loss-avoidance [78]. However, changes in frontostriatal connectivity with pharmacological treatment have not been adequately studied nor directly linked to positive symptom or cognitive improvement. More studies will be needed to experimentally test whether faulty frontostriatal coupling is indeed related to the expression of positive symptoms and whether drug treatment, with its dopaminergic actions, are sufficient in correcting abnormal interactions between the striatum and prefrontal cortex.

Thalamic dysconnectivity with the cortex has been suggested to underlie sensory gating deficits which have been observed in schizophrenia [80]. Studies of thalamo-cortical connectivity in early schizophrenia or high-risk groups are lacking. However, reduced thalamo-frontal connectivity was found in chronic schizophrenia patients in a seed-based correlation study of resting state fMRI [81]. Reduced functional connectivity might reflect anatomical disturbances as has been shown in a diffusion-weighted MRI study using probabilistic tracking. Here, chronic schizophrenia patients were found to display reduced anatomical connectivity from the thalamus to the lateral prefrontal cortex which had implications on how patients performed on a working memory task and the associated brain activity [82]. One study [83] has looked at the effect of nicotine on smokers diagnosed with schizophrenia compared to otherwise healthy smokers, and examined functional connectivity during an auditory verbal working memory task. Using the thalamus as a seed region, the authors found that connectivity to the prefrontal cortex increased in smokers after nicotine administration and that this increase was exacerbated in schizophrenia patients. However, this study was conducted with a sample of medicated, chronic patients and antipsychotic medication has been found to increase the drive to smoke [84]. The nature of thalamic connectivity in non-chronic patient groups has yet to be determined in order to separate medication effects from inherent abnormalities in the connectivity of the thalamus to frontal lobe. 

Schizophrenia patients often display symptoms of social withdrawal and flattened affect. Interactions between the amygdala and prefrontal cortex are thought to be important in emotional processing and generation of context-appropriate emotional response [85]. Therefore, in schizophrenia, fronto-amygdala connectivity has often been studied in relation to negative symptom expression and with social cognition tasks which are thought to probe them [86]. The fronto-amygdala circuit has also been found abnormal in resting state studies of schizophrenia [87] and first-degree relatives [88]. Currently, there are no studies which have looked at connectivity changes with drug treatment or challenges in schizophrenia. One study suggests that treatment with the SGA, olanzapine, might change fronto-amygdala connectivity. Blasi *et al.* [31] studied brain activity of patients during the processing of emotionally valent stimuli and found hyperactivity of amygdala compared to controls at four weeks of olanzapine treatment which decreased at eight weeks. Activity in ventrolateral prefrontal cortex, on the other hand, was found to be decreased in patients compared to controls at four weeks but it increased activity after eight weeks of treatment. Whether this inverse response of amygdala and ventrolateral prefrontal cortex to olanzapine treatment was associated with a change in functional connectivity between the two regions has not been explicitly addressed. 

## DYSCONNECTIVITY IN THE CORTICOTHALAMO-CEREBELLAR CIRCUIT

Andreasen *et al.* [89] has proposed a dysconnectivity theory which involves the cerebellum to explain the cognitive disturbances found in schizophrenia. This ’cognitive dysmetria’ model suggests that the cortico-thalamo-cerebellar circuit in schizophrenia is disrupted and that this leads to the perceptual, cognitive and behavioural disturbances found in schizophrenia. A recent resting state study found that the cerebellum was functionally dysconnected with subcortical regions including the thalamus as well as with cortical regions including the inferior frontal lobe in first-episode schizophrenia patients compared to healthy control subjects [90]. Interestingly, the authors found that unaffected siblings did not display similar dysconnectivity between the cerebellum and thalamus which might suggest that this particular dysconnectivity is a state rather than trait feature of the illness or perhaps a consequence of drug treatment.

In a study with schizophrenia patients observing changes of the cortico-thalamo-cerebellar circuit from a three-week treatment period with olanzapine, Stephan *et al.* [91] found that patients (n=6) exhibited increased functional connectivity between the cerebellum and the medial prefrontal regions as well as decreased connectivity between cerebellum and the mediodorsal thalamus after olanzapine treatment, which was also assessed to be significantly decreased compared to control subjects (n=6). Decreased connectivity to the cerebellum has also been found in a study that used structural equation modelling to assess effective connectivity within the cortico-thalamo-cerebellar network. Schizophrenia patients compared to control subjects exhibited decreased prefronto-cerebellar and cerebellar-thalamus connectivity as well as an (perhaps compensatory) increased thalamo-frontal connectivity during a working memory task [92]. Abnormal connectivity was more pronounced in schizophrenia patients medicated with FGAs (n=6) compared with those treated with SGAs (n=6), which could suggest a greater ameliorating, or less of a detrimental, effect of SGAs on prefronto-cerebellar and cerebellar-thalamus connectivity compared to FGAs. Future longitudinal studies examining connectivity before and after treatment with SGAs could clarify whether SGAs are beneficial for cerebellar connectivity or merely the ‘lesser of two evils’.

## DISRUPTED CORTICO-CORTICAL CONNECTIVITY

A fronto-temporal dysconnectivity was first reported by Friston and Frith [43] who found that during a word generation task the relationship between activity of the lateral prefrontal and temporal cortices in healthy controls was negative but in schizophrenia patients this relationship was positive. These findings have been replicated in a study which used dynamic causal modelling of fronto-temporal interactions during working memory of first-episode patients and in individuals in at-risk mental state of developing psychosis [73]. Yet a regional cerebral blood flow (rCBF) PET study on verbal fluency in obligate carriers of genetic risk for schizophrenia spectrum disorder (unaffected individuals with a child and a parent diagnosed with schizophrenia; n=10) and patients with schizophrenia in remission (n=10) found no evidence for fronto-temporal dysconnectivity [93]. This inconsistency could be due to a difference in the level of overt symptom expression between at-risk individuals or first-episode patients relative to unaffected relatives or stable patients. 

In other studies, aberrant connectivity between the prefrontal and temporal lobes in chronic medicated patients has been found across a wide range of cognitive tasks and has been implicated in positive symptom expression, in particular, auditory hallucinations [9, 94-97]. In the healthy brain, interaction between cortical systems allows for normal attribution of self-generated thoughts and actions as a result of what is called corollary discharge. A disintegration of this function in schizophrenia might explain the misattribution of internally generated speech leading to auditory hallucinations and other perceptual disturbances which are related to fronto-temporal dysconnectivity findings [98]. The association with positive symptoms is somewhat supported by a diffusion-weighted MRI study [99], where increased widespread diffusivity in the white matter of medication-free schizophrenia patients compared to healthy controls was observed. These patients were subsequently treated with antipsychotics for four weeks and divided into whether they improved on positive symptoms or not. Those who responded to antipsychotic treatment after four weeks showed decreased diffusivity in the left temporal lobe and cingulate gyrus as well as the right pyramidal tract, which at baseline were abnormally increased in diffusivity compared to control subjects. Drug non-responders showed no improvement in white matter diffusivity. The findings suggest that antipsychotics increase the anisotropic diffusion within temporal lobe white matter, thereby strengthening connectivity. This might underlie the improvement of positive symptoms in patients who respond to treatment. 

Fletcher *et al.* [95] found a suppressive effect of the activity in anterior cingulate and prefrontal cortices on the superior temporal cortex in healthy control subjects, but not in schizophrenia patients. In a separate rCBF-PET study, untreated schizophrenia patients were found to increase anterior cingulate activity during a verbal fluency task after acute administration of the dopamine antagonist apomorphine with a trend-level effect on the regulation of prefrontal and temporal interactions [100]. In a resting state fMRI study, fronto-temporal connectivity, as assessed by independent component analysis, was increased in schizophrenia patients compared to controls at baseline and subsequently normalised in patients after six weeks of antipsychotic treatment [101]. These studies suggest that antipsychotic treatment might to some degree normalise fronto-temporal connectivity. The improved functional integration across these cortical areas might contribute to the beneficial effect of antipsychotics on positive symptoms.

Fronto-parietal interactions are associated with many cognitive tasks involving attention, working memory and executive function, all of which are deficient in schizophrenia patients [102]. First-episode schizophrenia patients have been found to exhibit reduced resting state connectivity between the dorsolateral prefrontal cortex and parietal cortex [103]. Also for working memory-related brain activity, fronto-parietal dysconnectivity has been revealed with rCBF-PET [104] and fMRI [105] in chronic schizophrenia patients. One study [92] which looked at effective connectivity during working memory, found connectivity from the left dorsolateral prefrontal region to the left parietal cortex to be increased in patients treated with FGAs compared to patients treated with SGAs along with increased connectivity between the ventrolateral and dorsolateral prefrontal cortex. It was speculated that increased prefrontal-parietal connectivity might compensate for the abnormal cerebello-subcortico-cortical interactions (mentioned in the previous section) which was found more pronounced in the FGA-treated patients than the SGA-treated patients. Alternatively, the increased connectivity could be due to direct modulations of FGAs on the working memory-related connectivity between the frontal and parietal regions. However, since antipsychotic drug treatment is relatively ineffective in alleviating cognitive deficits, it might have little direct impact on cognitive brain networks. Future studies would be needed to investigate whether indeed there is a change in fronto-parietal connectivity with treatment and, if so, whether it is a primary or secondary effect.

## ALTERATIONS IN NETWORK CHARACTERISTICS AND TOPOLOGY IN SCHIZOPHRENIA

The fronto-parietal network can also be identified in resting state neuroimaging data. Since it is activated during cognitive tasks and anti-correlated with the default mode network [106] it is also referred to as a ‘task-positive’ network. The default mode network consists of anterior and posterior midline cortical areas and has been associated with self-referential processing and ‘mind-wandering’ [107]. It is less active during performance on cognitive tasks as opposed to rest and has therefore also been labelled as the ‘task-negative’ network. Connectivity within the default mode network has been found disrupted in schizophrenia patients during task and rest. In an fMRI working memory study, chronic patients with schizophrenia displayed hyperconnectivity of the medial prefrontal cortex (a key node of the default mode network) to the rest of the default mode network [108]. The connectivity pattern of unaffected first-degree relatives lay between the pattern found in patients and healthy control subjects [108]. This study suggests that dysconnectivity of the default mode network is at least to some extent conferred by genetic risk and might be aggravated by the disease state. 

The increased functional coupling of the medial prefrontal regions and the default mode network might be modulated by antipsychotic medication. Indeed, a longitudinal fMRI study using a working memory task at four and eight weeks of olanzapine treatment found that prolonged treatment with olanzapine strengthened functional connectivity between ventromedial prefrontal cortex and the default mode network, but did not affect the connectivity strength of the posterior regions which had displayed altered connectivity with the default mode network in patients compared to healthy control subjects [109]. Greater connectivity of the ventromedial prefrontal cortex with the default mode network during a working memory task might indicate improved cognitive control over task-irrelevant networks promoting more efficient re-allocation of neural resources to task-relevant networks [110]. In a multi-site fMRI study of schizophrenia patients performing an auditory-motor task [111], SGA-medicated patients (n=72) tended to display stronger task-modulation of the task-related network and the default mode network than those receiving FGAs (n=7). SGAs might differ from their predecessors in their downstream effect on dopamine signalling in the prefrontal cortex which might directly modulate the default mode network *via *connections from the medial prefrontal cortex. GABA interneurons are also important components for prefrontal connectivity, mediating the firing of glutamatergic and dopaminergic neurons between the striatum and prefrontal cortex, and drugs acting on GABAergic receptors have also been found to modulate working memory performance and related brain network response in a differential manner in schizophrenia patients than in healthy control subjects [112]. Flumazenil, a GABA_A_ receptor partial agonist, was found to increase deactivations of the task-negative network during working memory performance in schizophrenia subjects (n=12), although it attenuated neural network response to task in control subjects (n=12). Behaviourally, flumazenil also improved task performance in the patient group but worsened it in the control group (thereby decreasing between-group performance differences).

However, the neuroimaging data on the effects of antipsychotic treatment on functional activity and connectivity in the default mode network is heterogeneous. For instance, it was shown that patients with schizophrenia still exhibited attenuated task-suppression of the medial prefrontal cortex during working memory despite being medicated and despite displaying increased connectivity to the rest of the default mode network compared to controls and unaffected relatives [110]. Another study examining resting state networks in antipsychotic-naïve schizophrenia patients before and after six weeks of antipsychotic treatment, did not find an ameliorating effect of antipsychotic treatment [101]. In this same study, the default mode network connectivity was also found decreased compared to control subjects, which is contrary to the hyperconnectivity found in the aforementioned activation studies. It should be further noted that several other resting state studies of chronic, medicated patients have found decreased, rather than increased, connectivity between regions of the default mode network e.g. medial prefrontal cortex, precuneus, posterior cingulate cortex, inferior parietal lobe and medial temporal regions [113-117]. Also, not all studies have found resting state or task-induced deactivation abnormalities of the default mode network [118, 119]. In general, the literature surrounding default mode network dysconnectivity in schizophrenia patients seems to be inconsistent [120].

Distributed patterns of functional brain connectivity have also been examined using measures which characterise each node in brain networks in relation to other network nodes. For instance, topological features of brain connectivity can be described with graph theory by estimating the number of connections a node has or the length one node takes to reach another which determines how costly a network is to maintain and use (Fig. **1c**) [121]. It has been shown that the functional architecture of brain connectivity has ‘small world’ properties that minimise costs and maximise efficiency in interregional signal transmissions [122]. Graph theoretical analyses of fMRI data have revealed a disruption of the small-world network in the schizophrenic brain including those of first-episode patients [123-126] as well as high-risk individuals [127]. This has been interpreted as evidence for inefficient neural integration in functional brain networks. This costliness of brain network activation might explain the lower cognitive capacities of schizophrenia patients [128]. Effects of antipsychotic treatment in patients have yet to be investigated although in healthy and aging subjects global and local efficiency of brain networks have been found detrimentally affected with an acute dose of sulpiride, a selective D_2_ receptor antagonist [129]. In one study with schizophrenia patients, the disruption of small-world properties correlated with the chlorpromazine-equivalent dose patients were receiving [123]. But, as Fornito *et al.* [40] have pointed out, longitudinal studies charting changes in graph theoretic measures are warranted in order to properly investigate drug treatment-related changes in functional brain topology in psychosis patients.

## FUTURE PERSPECTIVES

Connectivity differences between patients with schizophrenia and healthy control subjects seem to be widespread and involving many of the interactions that are important for efficient information processing. However, the bulk of the neuroimaging literature in schizophrenia is merely observational, assessing brain activity and connectivity many years after the neurodevelopmental changes that lead to the illness-onset. Longitudinal neuroimaging research of high-risk populations is therefore warranted to identify causal relationships between brain connectivity, illness, and treatment. The published studies on longitudinal changes in brain connectivity before and after antipsychotic treatment are very few and some suffer from small sample sizes (see Table **1** for a summary) and the modulatory effects of antipsychotic drug treatment on functional connectivity of subcortico-cortical circuits and whole-brain functional topology remain to be thoroughly investigated. Other aspects of drug-induced changes in functional brain connectivity, for example, the drug effects on cerebellar-cortical, fronto-temporal, and fronto-parietal local circuits have so far only been rudimentarily explored.

Another relevant question that remains to be answered is how much does functional dysconnectivity represent a state or trait marker of schizophrenia spectrum disorder. There is neuroimaging evidence to suggest that frontostriatal and fronto-temporal dysconnectivity might be state features related to positive symptom expression. Some other alterations are already evident in the early phases of psychosis or even in subjects at high genetic risk for the illness, suggesting that some dysconnectivity could represent a stable neurobiological core feature of psychosis. However, findings are not always consistent. Not only do the areas of disturbed connectivity differ across studies but so does the direction of change, i.e. whether disturbances reflect increased or decreased connectivity. These inconsistencies might reflect differences in psychopathology or analytical methods. Many methodological factors may influence the observed changes in connectivity. The state of the brain (task vs. rest), the emotional and cognitive processes activated by a given task, differences in task performance between patients and control subjects [126], and the way the data is pre-processed and analysed [130] may have substantial impact on the connectivity patterns and, thus, need to be taken into account when interpreting the observed dysconnectivity patterns. The heterogeneity of patient groups poses additional problems, as some subgroups may present with a different pattern of dysconnectivity relative to other subgroups. Future studies might therefore consider stratifying patients more stringently according to clinical profiles and response to medication since a lot of important information is potentially lost in the group averaging of imaging and clinical data. 

There is also a need for studies to determine the causal relationship between inter-connected regions. Due to its poor temporal resolution, causal relationships can be difficult to assess with fMRI. The combination of EEG and fMRI offers the temporal resolution of EEG as well as the high spatial resolution of fMRI and potentially allows for a more sophisticated assessment of the inter-regional direction of information flow [131]. Effective connectivity analyses such as dynamic causal modelling have also proved useful in modelling the influence of one region’s activity on another and the modulatory effects of drugs on these causal connections [132]. Combining transcranial magnetic stimulation (TMS) and fMRI [59] is also another promising approach in assessing the direction of connectivity between brain structures in schizophrenia [133]. TMS can be used to focally perturb the activity of a distinct cortical region as well as its connections with remote brain areas. As such TMS offers a means of experimentally manipulating distinct brain connections, for instance, those that are dysfunctional in schizophrenia. 

Another point to consider when interpreting abnormal connectivity patterns is whether alterations in brain connectivity result from regionalised deficits that reverberate across networks [134, 135]. Alternatively, dysconnectivity patterns could be due to a deficient axonal transmission and information exchange among otherwise normally functioning brain regions. One way to address this would be to link abnormal anatomical connectivity to functional dysconnectivity in schizophrenia by combining diffusion-weighted MRI and fMRI [117, 136, 137]. Findings seem to point to functional dysconnectivity not fully explained in terms of anatomical dysconnectivity although this is as yet inconclusive.

The studies of brain connectivity changes in schizophrenia and its modification by drug treatment might ultimately help the development of effective interventions. If one assumes that dysconnectivity underlies the expression of symptoms and can be pharmacologically targeted, drug-induced changes in functional brain connectivity might provide a useful imaging marker that might inform the development of new drugs. Considering the complexity and heterogeneity of schizophrenia, it would be somehow naïve to assume that a single pharmaceutical compound will be able to globally modify all aspects of pathogenetic dysconnectivity in schizophrenia [138]. A more realistic scenario is that a specific compound might target a specific aspect of abnormal brain connectivity and hereby improve a specific cognitive deficit or a specific clinical symptom. Also, some dysconnectivity might actually be compensatory and therefore beneficial in which case antipsychotic treatment should reinforce this ‘abnormal’ connectivity pattern. Such considerations emphasises the importance of relating dysconnectivity to behavioural and clinical outcomes in future studies.

If not to identify potential targets for drug development, an increased effort in prospectively assessing the changes of connectivity with drug challenges or treatment would be as importantly helpful in distinguishing drug-induced from disease-induced changes in brain activity and connectivity. The vast majority of research into schizophrenia is conducted on already-treated, chronic patients and there is a need to identify drug confounds from abnormalities inherent to the disorder in order for treatment options and understanding to progress. 

## Figures and Tables

**Table 1.** Summary of fMRI Studies of Connectivity with Antipsychotic Treatment in Schizophrenia*

Study	Subjects	Pharmacological Intervention and Design	Task and Analysis Method	Main Findings
Abbott *et al.* (2011) [112]	79 chronic schizophrenia patients, 114 healthy control subjects.	7 treated with FGAsa; 72 treated with SGAsb. Cross-sectional.	Auditory-motor task. Independent component analysis (beta-weights of regression to task).	Task-modulation of task and default mode networks decreased in FGA^b^- compared to SGA^a^-treated patients and with higher doses.
Lui *et al.* (2010) [102]	34 antipsychotic--naïve, first-episode schizophrenia patients, 34 healthy control subjects.	SGA^b^ treatment. Longitudinal: scanned before and after 6 weeks treatment.	Resting-state. Seed-voxel correlation and independent component analysis.	Fronto-temporal hyperconnectivity in patients compared to control subjects at baseline; normalised after treatment. No change in default mode network connectivity which was increased at baseline in patients compared to controls.
Sambataro *et al.* (2010) [110]	17 schizophrenia patients (13 medication-naïve and 6 medication-free), 19 healthy control subjects.	Olanzapine monotherapy. Longitudinal: scanned at 4 and 8 weeks of treatment.	N-back working memory task. Independent component analysis.	Default mode connectivity increased in precuneus and inferior parietal lobe, and decreased in posterior cingulate cortex in patients compared to controls. Increased connectivity with treatment of the medial prefrontal cortex to the default mode network in patients.
Schlosser *et al.* (2003) [93]	12 chronic schizophrenia patients, 6 healthy control subjects.	6 SGA^b^-treated (5 with olanzapine, 1 with quetiapine); 6 FGA^a^-treated with haloperidol. Cross-sectional.	N-back working memory task. Structural equation modelling of cerebello-subcortico-cortical effective connectivity.	Schizophrenia patients displayed decreased prefronto-cerebelllar and cerebello-thalamus connectivity, and increased thalamo-prefrontal connectivity compared to controls. Dysconnectivity more pronounced in FGA^a^-treated compared to SGA^b^-treated patients. Increased connectivity from left dorsolateral prefrontal cortex to left parietal cortex and between ventrolateral and dorsolateral prefrontal cortex in patients treated with FGA^a^ compared to SGA^b^-treated patients.
Stephan *et al*. (2001) [92]	6 patients medication-free (4 drug-naïve; 2 previously FGA^a^-treated), 6 healthy control subjects.	Olanzapine monotherapy. Longitudinal: scanned before and after 3 weeks treatment.	Finger-tapping task. Seed-voxel correlation analysis.	Decreased cerebellar connectivity with mediodorsal thalamus and increased cerebellar connectivity with lateral and medial prefrontal cortex after treatment. Tendency for connectivity to normalise after treatment.

* Studies were selected if they: i) included a psychosis patient sample, ii) investigated antipsychotic treatment effects, iii) with fMRI, and iv) analysed imaging data with functional or effective connectivity methods.

a FGAs = first generation antipsychotics

b SGAs = second generation antipsychotics

## ABBREVIATIONS

BOLD: = Blood oxygenation level dependent
EEG: = Electroencephalography
FGAs: = First generation antipsychotics
fMRI: = Functional magnetic resonance imaging
GABA: = Gamma-aminobutyric acid
MEG: = Magnetoencephalography
MRI: = Magnetic resonance imaging
NMDA: = N-methyl-D-aspartate
PET: = Positron emission tomography
rCBF: = Regional cerebral blood flow
SGAs: = Second generation antipsychotics
TMS: = Transcranial magnetic stimulation
